# ﻿﻿﻿Three novel species and new records of *Kirschsteiniothelia* (Kirschsteiniotheliales) from northern Thailand

**DOI:** 10.3897/mycokeys.101.115286

**Published:** 2024-02-02

**Authors:** Antonio Roberto Gomes de Farias, Naghmeh Afshari, Veenavee S. Hittanadurage Silva, Johnny Louangphan, Omid Karimi, Saranyaphat Boonmee

**Affiliations:** 1 Center of Excellence in Fungal Research, Mae Fah Luang University, Chiang Rai, 57100, Thailand Mae Fah Luang University Chiang Rai Thailand; 2 Department of Biology, Faculty of Sciences, Chiang Mai University, Chiang Mai 50200, Thailand Mae Fah Luang University Chiang Mai Thailand; 3 School of Science, Mae Fah Luang University, Chiang Rai 57100, Thailand Chiang Mai University Chiang Rai Thailand

**Keywords:** Multilocus phylogeny, new host records, saprobic fungi, three new species, woody litter

## Abstract

*Kirschsteiniothelia* (Kirschsteiniotheliales, Pleosporomycetidae) includes 39 saprobic species recorded from dead or decaying wood in terrestrial and freshwater habitats. This study focuses on exploring *Kirschsteiniothelia* diversity in woody litter in Thailand. Wood samples were collected from forest areas in Chiang Rai and Chiang Mai Provinces in Thailand and examined for fungal fructifications. Fungal isolates were obtained and their morphological and sequence data were characterised. Micromorphology associated with multilocus phylogeny of ITS, LSU and SSU sequence data identified three isolates as novel species (*Kirschsteiniotheliainthanonensis*, *K.saprophytica* and *K.zizyphifolii*) besides new host records for *K.tectonae* and *K.xishuangbannaensis*. The placement of the new taxa and records are supported by morphological illustrations, descriptions and molecular phylogenies and the implications of these findings are discussed. Our findings provide information for understanding *Kirschsteiniothelia* diversity and ecology.

## ﻿Introduction

Since its introduction by Hawksworth (1985), the taxonomic placement of *Kirschsteiniothelia* (Kirschsteiniotheliaceae, Pleosporales, Pleosporomycetidae) has undergone several revisions. It was introduced in Pleosporaceae, with *Kirschsteiniotheliaaethiops* as the type species. However, Barr (1993) moved it to Pleomassariaceae based on morphology and, based on molecular phylogenetic analyses, Schoch et al. (2006) demonstrated that *K.aethiops* does not belong to Pleosporaceae and should be placed in a new family. Kirschsteiniotheliaceae was established by Boonmee et al. (2012) to accommodate the holomorphic genus *Kirschsteiniothelia*. This was due to the fact that *K.elaterascus* and *K.maritima* clustered into Morosphaeriaceae and Mytilinidiales, respectively (Schoch et al. 2009; Suetrong et al. 2009; Boonmee et al. 2012). Later, Hernández-Restrepo et al. (2017) assigned it to the newly-proposed order Kirschsteiniotheliales (Dothideomycetes) due to its phylogenetic significance. Boonmee et al. (2012) also synonymised *Dendryphiopsisatra* under *K.atra* (Corda) D. Hawksw. due to their phylogenetic and asexual morph similarity (Boonmee et al. 2012; Schoch et al. 2009). The placement of *Kirschsteiniothelia* in the latest Outline of fungi and fungus-like taxa (Wijayawardene et al. 2022) is Kirschsteiniotheliaceae, Kirschsteiniotheliales, Dothideomycetes order incertae sedis, Dothideomycetes, Ascomycota.

*Kirschsteiniothelia* sexual morphs essentially have superficial to semi-immersed, subglobose to globose, dark brown to black ascomata; cylindrical clavate, bitunicate, 8-spored asci; and brown to dark brown, ellipsoidal, septate ascospores with or without a mucilaginous sheath (Hawksworth 1985; Boonmee et al. 2012; Hyde et al. 2013). However, its asexual morphs include dendryphiopsis-like and sporidesmium-like structures, with *Dendryphiopsis* taxa confirmed to be linked to *Kirschsteiniothelia*, based on morphology and molecular evidence (Schoch et al. 2009; Boonmee et al. 2012).

*Kirschsteiniothelia* species are mostly saprobes on dead or decaying wood in freshwater and terrestrial habitats (Boonmee et al. 2012; Hyde et al. 2013; Su et al. 2016; Mehrabi et al. 2017; Bao et al. 2018; Dong et al. 2020; Sun et al. 2021; Liu et al. 2023). These taxa play a crucial role in nutrient cycling and decomposition processes, contributing to the breakdown of organic matter in their respective ecosystems (Bucher et al. 2004). Their ability to colonise wood in freshwater habitats further emphasises their ecological significance (Su et al. 2016). In addition, Nishi et al. (2018) reported *Kirschsteiniothelia* associated with ankle bursitis in a Japanese patient and Guegan et al. (2021) with foot chromoblastomycosis in an immunosuppressed patient. Besides, Poch et al. (1992) discovered new compounds in *Kirschsteiniothelia* species, including kirschsteinin, which showed antimicrobial activity and Bugni and Ireland (2004) reported antibacterial activity from *K.maritima*.

This study focuses on exploring *Kirschsteiniothelia* diversity in woody litter in Thailand. We introduce three new species viz. *K.inthanonensis*, *K.saprophytica* and *K.zizyphifolii*, along with two new host records of *Kirschsteiniothelia*, based on a morpho-molecular approach, expanding our knowledge of the diversity in Pleosporomycetidae.

## ﻿Material and methods

### ﻿Sample collection, fungal isolation and microscopic characterisation

Wood litter samples were collected from forest areas in Chiang Rai and Chiang Mai, Thailand. Morphological studies were performed following the methods described by Senanayake et al. (2020). The fungal structures were examined using a Leica EZ4 stereomicroscope. The micro-morphological features were observed and photographed using a Nikon ECLIPSE Ni compound microscope with a Canon 600 D digital camera. The Tarosoft Image Frame Work programme was used to measure specimen structures, and photo plates were prepared using the open-source Inkscape v.1.3 (https://inkscape.org/).

Pure cultures were obtained through single spore isolation on Difco potato dextrose agar (PDA) using the spore suspension method (Choi et al. 1999). Germinating spores were transferred to a new PDA plate and incubated at room temperature for seven days. Ex-type pure living cultures were deposited in the Mae Fah Luang University Culture Collection (MFLUCC) and herbarium material was deposited in the Mae Fah Luang University Fungarium (MFLU), Chiang Rai, Thailand. Faces of fungi numbers (FoF) (Jayasiri et al. 2015) and Index Fungorum numbers (Index Fungorum 2023) were obtained as instructed and the data were uploaded to the Greater Mekong Subregion in the GMS database (Chaiwan et al. 2021).

### ﻿DNA extraction, PCR amplification and sequencing

Genomic DNA was extracted from fresh mycelium scrapings using the EE.Z.N.A. Tissue DNA Kit from Omega Bio-tek, Inc., following the manufacturer’s instructions. PCR amplifications were performed in a 50 μl reaction volume containing 10× PCR Master Mix, forward and reverse primers, DNA template and double sterilised H_2_O. Amplified DNA of the ITS, LSU and SSU were obtained through a polymerase chain reaction (PCR) using the pairs of primers ITS4/ITS5 (White et al. 1990), LROR/LR5 (Vilgalys and Hester 1990) and NS1/NS4 (White et al. 1990), correspondingly. The quality of the PCR products was visualised on a 1% agarose gel and sequenced by Biogenomed Co., Ltd (South Korea).

### ﻿Alignments and phylogenetic analyses

The reads were assembled using the Staden Package (Staden et al. 2003) and compared against the NCBI non-redundant GenBank database (Sayers et al. 2020) and related reference sequences downloaded (Table 1). Except for concatenation and visualisation, all the steps of phylogenetic analysis were conducted in a Windows Subsystem for Linux (Microsoft, USA). The individual datasets were aligned using MAFFT with the --*auto* flag and automatically trimmed using TrimAl v.1.3 with the -*gt* (0.3) option (Capella-Gutierrez et al. 2009). The best-fit model was selected using ModelTest-NG v.0.1.7 with the --*template mrbayes* option for DNA 3 schemes matrices (Darriba et al. 2020). The alignments were concatenated using SequenceMatrix and subjected to Maximum Likelihood (ML) and Bayesian Inference (BI) analyses.

**Table 1.** Names, strain numbers, and corresponding GenBank accession numbers of *Kirschsteiniotheliales* taxa used in the phylogenetic analyses.

Taxa	Strains	Accession numbers		
		ITS	LSU	SSU
*Acrospermumadeanum*	M133	EU940180	EU940104	EU940031
*Acrospermumcompressum*	M151	EU940161	EU940084	EU940012
*Acrospermumgramineum*	M152	EU940162	EU940085	EU940013
*Aliquandostipitecrystallinus*	R 76–1	–	EF175651	EF175630
*Aliquandostipitekhaoyaiensis*	CBS 118232^T^	–	GU301796	–
*Anisomeridiumubianum*	MPN94	–	GU327709	JN887379
*Dyfrolomycesrhizophorae*	JK5456A	–	GU479799	GU479766
*Dyfrolomycestiomanensis*	NTOU3636	–	KC692156	KC692155
*Flavobatheliumepiphyllum*	MPN67	–	GU327717	JN887382
*Halokirschsteiniotheliamaritima*	CBS 221.60	–	AY849943	AF053726
*Helicomycesroseus*	CBS 283.51	AY916464	AY856881	AY856928
	MFLUCC 15–0343	KY320523	KY320540	–
*Homortomycescombreti*	CPC 19808^T^	JX517281	JX517291	–
*Homortomycestamaricis*	MFLUCC 13–0280	KU752184	KU561874	KU870905
	MFLUCC 14–0167	KU934190	KU561875	–
	MFLUCC 13–0441^T^	NR_155161	NG_059495	–
*Jahnulabipileata*	F49–1 ^T^	JN942353	EF175657	EF175635
*Jahnulasangamonensis*	A402–1B	JN942349	EF175661	EF175639
*Jahnulaseychellensis*	SS 2113.2	–	EF175664	EF175643
*Kirschsteiniotheliaacutispora*	MFLU 21–0127^T^	OP120780	ON980758	ON980754
*Kirschsteiniotheliaaquatica*	MFLUCC 16–1685^T^	MH182587	MH182594	MH182618
*Kirschsteiniotheliaarasbaranica*	IRAN 2509C	KX621986	KX621987	KX621988
	IRAN 2508C^T^	KX621983	KX621984	KX621985
*Kirschsteiniotheliaatra*	DEN	MG602687	–	–
	CBS 109.53	–	AY016361	AY016344
	MFLUCC 16–1104	MH182583	MH182589	MH182615
	S–783	MH182586	MH182595	MH182617
	MFLUCC 15–0424	KU500571	KU500578	KU500585
*Kirschsteiniotheliacangshanensis*	GZCC19–0515	–	MW133829	MW134609
	MFLUCC 16–1350^T^	MH182584	MH182592	–
	MFLU 23–0358^T^	OR575473	OR575474	OR575475
*Kirschsteiniotheliacrustaceum*	MFLU 21–0129^T^	MW851849	MW851854	–
*Kirschsteiniotheliadushanensis*	GZCC 19–0415	OP377845	MW133830	MW134610
*Kirschsteiniotheliaebriosa*	CBS H–23379	–	LT985885	–
*Kirschsteiniotheliaemarceis*	MFLU 10–0037^T^	NR_138375	NG_059454	–
*Kirschsteiniotheliaextensum*	MFLU 21–0130^T^	MW851850	MW851855	–
*Kirschsteiniotheliafluminicola*	MFLUCC 16–1263^T^	MH182582	MH182588	–
***Kirschsteiniotheliainthanonensis***	**MFLUCC 23–0277^T^**	**OR762773**	**OR762781**	**OR764784**
*Kirschsteiniothelialignicola*	MFLUCC 10–0036^T^	HQ441567	HQ441568	HQ441569
*Kirschsteiniothelianabanheensis*	HJAUP C2006	OQ023274	OQ023275	OQ023037
	HJAUP C2004^T^	OQ023197	OQ023273	OQ023038
*Kirschsteiniotheliaphoenicis*	MFLU 18–0153	NR_158532	NG_064508	–
	MFLUCC 18–0216^T^	MG859978	MG860484	MG859979
*Kirschsteiniotheliapuerensis*	ZHKUCC 22–0272	OP450978	OP451018	OP451021
	ZHKUCC 22–0271^T^	OP450977	OP451017	OP451020
*Kirschsteiniotheliarostrata*	MFLUCC 15–0619^T^	KY697280	KY697276	KY697278
*Kirschsteiniotheliaseptemseptatum*	MFLU 21–0126^T^	OP120779	ON980757	ON980752
***Kirschsteiniotheliasaprophytica***	**MFLUCC 23–0275 ^T^**	**OR762774**	**OR762783**	–
	**MFLUCC 23–0276**	**OR762775**	**OR762782**	–
*Kirschsteiniotheliaspatiosum*	MFLU 21–0128^T^	–	OP077294	ON980753
*Kirschsteiniotheliasubmersa*	S–481	–	MH182591	MH182616
	S–601	MH182585	MH182593	–
	MFLUCC 15–0427^T^	KU500570	KU500577	KU500584
*Kirschsteiniotheliatectonae*	MFLUCC 12–0050	KU144916	KU764707	–
	MFLUCC 13–0470	KU144924	–	–
***Kirschsteiniotheliatectonae***	**MFLUCC 23–0271**	**OR762771**	**OR762779**	**OR764782**
	**MFLUCC 23–0272**	**OR762772**	**OR762780**	**OR764783**
*Kirschsteiniotheliathailandica*	MFLUCC 20–0116^T^	MT985633	MT984443	MT984280
*Kirschsteiniotheliathujina*	JF13210	KM982716	KM982718	KM982717
*Kirschsteiniotheliavinigena*	CBS H–23378^T^	–	NG_075229	–
*Kirschsteiniotheliaxishuangbannaensis*	ZHKUCC 22–0221	OP289563	OP289565	OP303182
	ZHKUCC 22–0220^T^	OP289566	OP289564	OP303181
***Kirschsteiniotheliaxishuangbannaensis***	**MFLUCC 23–0273**	**OR762770**	**OR762778**	**OR764781**
	**MFLUCC 23–0274**	**OR762769**	**OR762777**	**OR764780**
***Kirschsteiniotheliazizyphifolii***	**MFLUCC 23–027^T^**	**OR762768**	**OR762776**	**OR764779**
*Megalotremisverrucosa*	MPN104	–	GU327718	JN887383
*Phyllobatheliumanomalum*	MPN 242	–	GU327722	JN887386
*Stemphyliumvesicarium*	CBS 191.86	MH861935	GU238160	GU238232
	MFLUCC 14–0920	KY659560	KY659563	KY659567
*Tubeufiahelicomyces*	CBS 271.52	AY916461	AY856887	AY856933
*Tubeufiajavanica*	MFLUCC 12–0545^T^	KJ880034	KJ880036	KJ880035
*Acrospermumadeanum*	M133	EU940180	EU940104	EU940031
*Acrospermumcompressum*	M151	EU940161	EU940084	EU940012

The newly-generated sequences are indicated in bold. ^“T”^ refers to holotype or ex-type strains and “–” shows unavailable data in GenBank.

Maximum Likelihood (ML) trees were generated using RAxML-HPC2 on XSEDE (8.2.8) (Stamatakis 2014) in the CIPRES Science Gateway platform (Miller et al. 2010), using 1,000 bootstraps replications and applying a partitioned model of evolution calculated by ModelTest-NG. Bayesian Inference was performed using MrBayes (Ronquist et al. 2012), with four simultaneous Markov Chain Monte Carlo (MCMC) chains and four runs for 3,000,000 million generations, sampling trees every 300^th^ generation. The first 25% of trees were discarded as burn-in and posterior probabilities (PP) were calculated from the remaining trees. The consensus phylograms were visualised using FigTree (Rambaut 2012) and edited using the open-source Inkscape v.1.3 (https://inkscape.org/).

## ﻿Results

### ﻿Phylogenetic analyses

**Figure 1.**
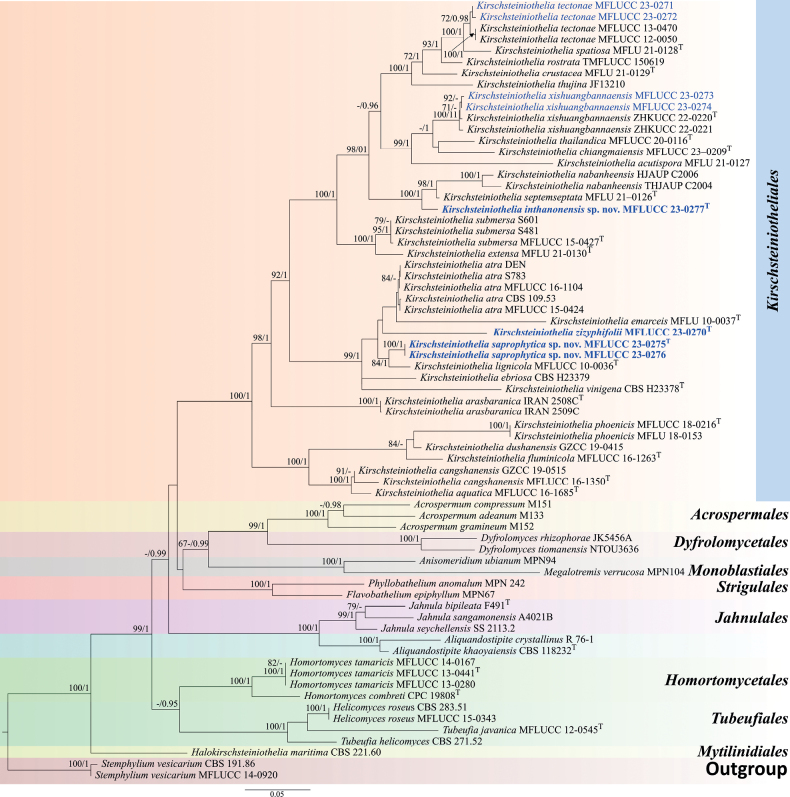
Maximum Likelihood phylogenetic tree generated from ITS, LSU and SSU sequence data for selected Kirschsteiniotheliales and related Dothideomycetes orders. The tree is rooted with *Stemphyliumvesicarium* (CBS 191.86 and MFLUCC 14–0920). Newly-generated sequences are in blue and new species are in bold. Holotype and ex-type strains are symbolic by “^T^”. Maximum Likelihood bootstrap (MLBS) values ≥ 70% and Bayesian posterior probabilities (BYPP) ≥ 0.95 are shown at the nodes.

The concatenated nucleotide alignment of the ITS, LSU and SSU datasets comprised 69 *Kirschsteiniotheliales* strains, including the outgroups (*S.vesicarium*MFLUCC 14–0920 and CBS191.86) and included 2,640 sites (ITS = 1–561; LSU = 562–1596; SSU = 1597–2640), of which 1,550 comprised of distinct alignment patterns (ITS = 427, LSU = 668 and SSU = 455), with of 32.01% undetermined characters or gaps. The final GAMMA-based score of the best tree was -24775.722822. Maximum Likelihood phylogeny and Bayesian analyses of single- and multi-loci had similar topologies and are combined in Fig. 1. Parameters for the models of each amplicon were described in Table 2. The Bayesian analysis tracer of the combined runs checked at six million generations had an effective sampling size for all the parameters higher than 3,000 and convergence diagnostic (PSRF = Potential Scale Reduction Factor; Gelman and Rubin (1992) of 1.0. The run resulted in 10,001 trees, of which 7,501 were sampled after 25% of the trees were discarded as burn-in. The alignment contained 1,802 unique sites (ITS = 427, LSU = 782, SSU = 593). The ML and BI analyses showed similar tree topologies.

**Table 2.** Maximum Likelihood indices of *Kirschsteiniothelia* tree.

Parameters	ITS	LSU	SSU
Evolutionary model	GTR+I+G4	GTR+G4	GTR+I+G4
Gamma distribution shape parameter α	0.267050	0.557118	0.228478
Estimated base frequencies			
A	0.199482	0.235780	0.260410
C	0.306708	0.238788	0.213687
G	0.279166	0.322010	0.267932
T	0.214644	0.203422	0.257971
Substitution rates			
AC	1.269939	0.865025	1.305091
AG	2.734587	2.259149	2.368982
AT	1.504952	1.054098	0.620257
CG	1.112253	0.931891	0.757010
CT	3.835090	5.793263	8.684577
GT	1.000000	1.000000	1.000000

**Figure 2.**
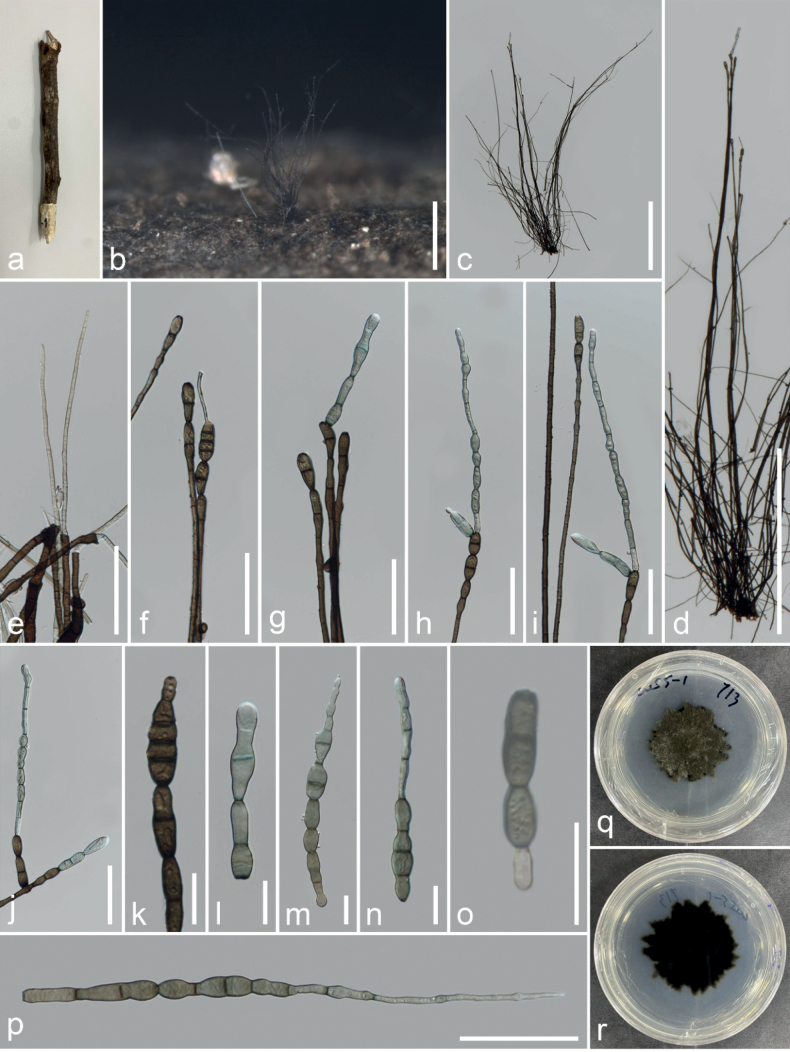
*Kirschsteiniotheliainthanonensis* (MFLU 23–0420, holotype) **a, b** colonies on the host **c, d** conidiophores and conidia **e** regeneration of conidiophores **f–j** conidiogenous cells and conidia **l–o** conidia **p** germinating conidium **q, r** colony on PDA (front and reverse). Scale bars: 500 μm (**b–d**); 50 μm (**e–j**); 20 μm (**k–p**).

**Figure 3.**
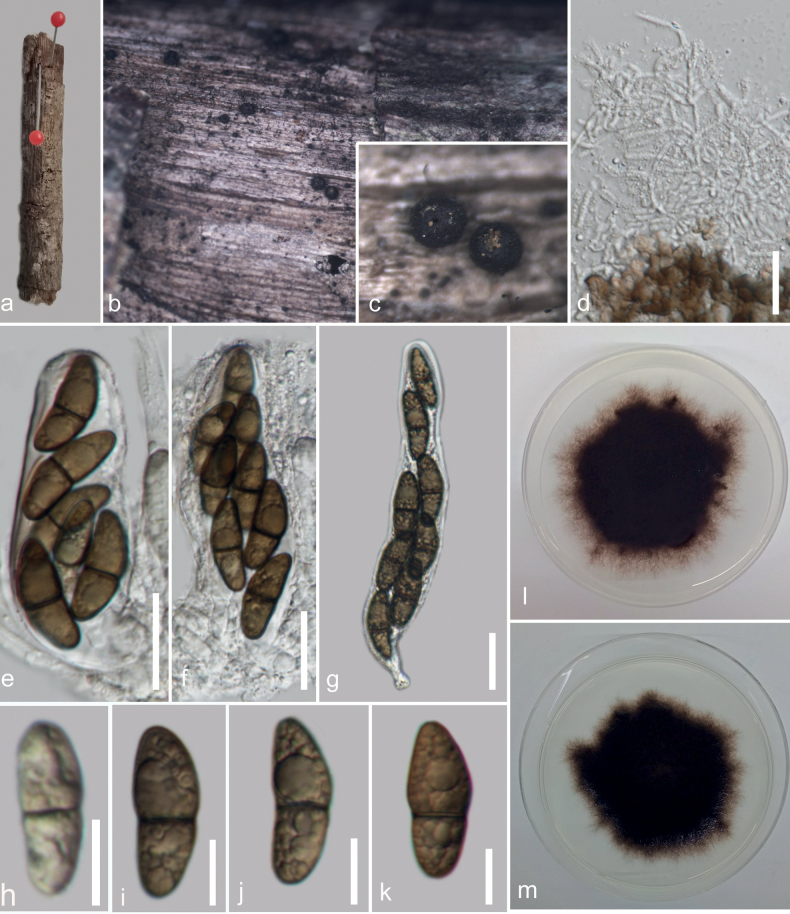
*Kirschsteiniotheliasaprophytica* (MFLU 23–0419, holotype) **a** host **b, c** appearance of ascomata on host surface **d** paraphyses **e–g** asci **h–k** ascospores **l, m** culture on PDA (front and reverse). Scale bars: 20 µm (**d–g**); 10 µm (**h–k**).

**Figure 4.**
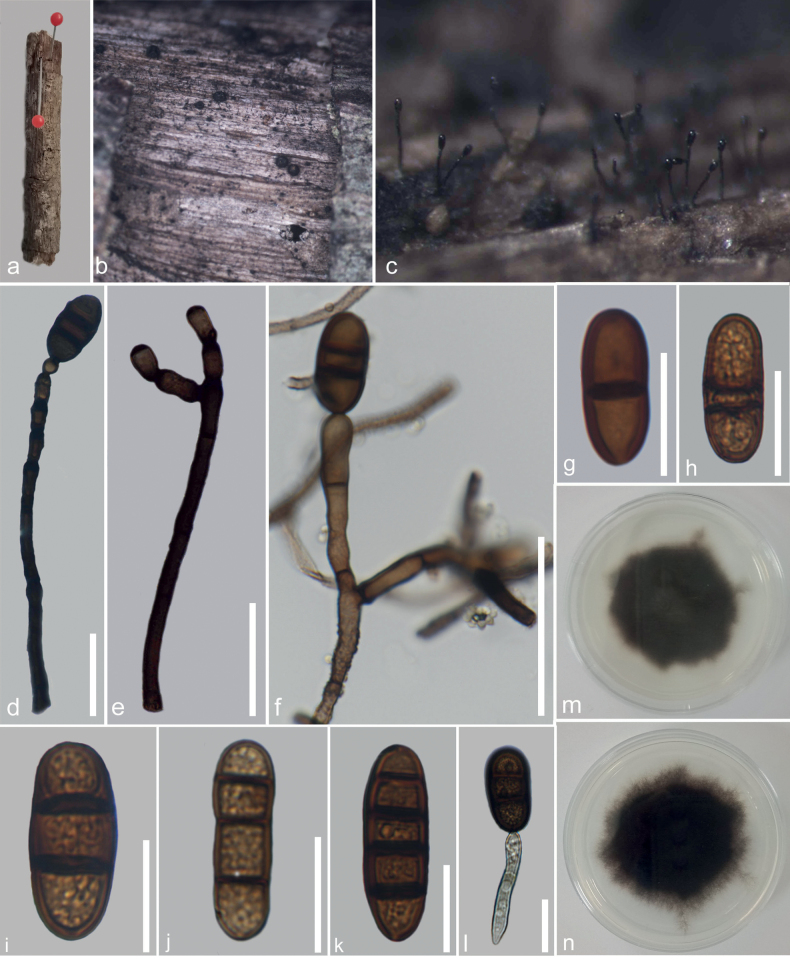
*Kirschsteiniotheliasaprophytica* (MFLUCC 23–0276) **a** host **b** colonies on the host, associated with asexual morph **c** conidiophore with conidiogenous cell and conidiospore **d, e** conidiophore (**e** – from the culture) **f–j** conidiospore from culture **k** germinating spore **m, n** culture on PDA (front and reverse). Scale bars: 50 µm (**c–e**); 20 µm (**f–k**).

**Figure 5.**
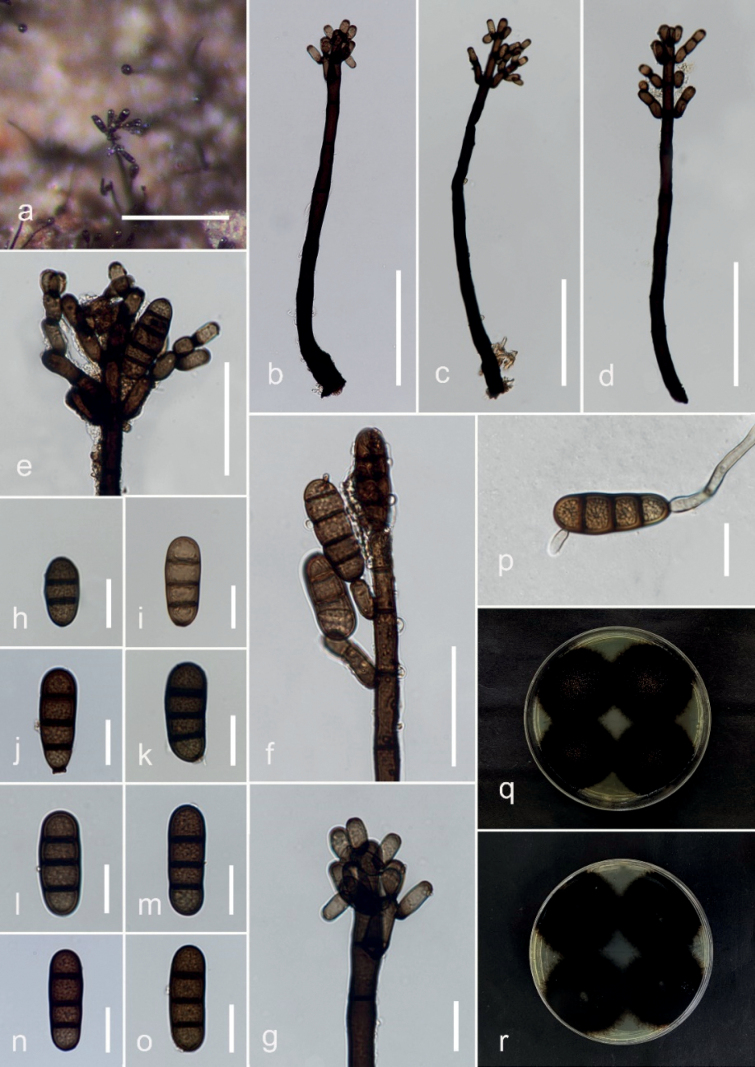
*Kirschsteiniotheliazizyphifolii* (MFLU 23–0415, holotype) **a** colonies on wood **b–d, g** conidiophores and conidiogenous cells **e, f** conidiophores with conidia **h–o** conidia **p** germinated conidium **q, r** cultures on PDA from the surface and reverse. Scale bars: 200 μm (**a**); 100 μm (**b–d**); 50 μm (**e, f**); 20 μm (**g–p**).

**Figure 6.**
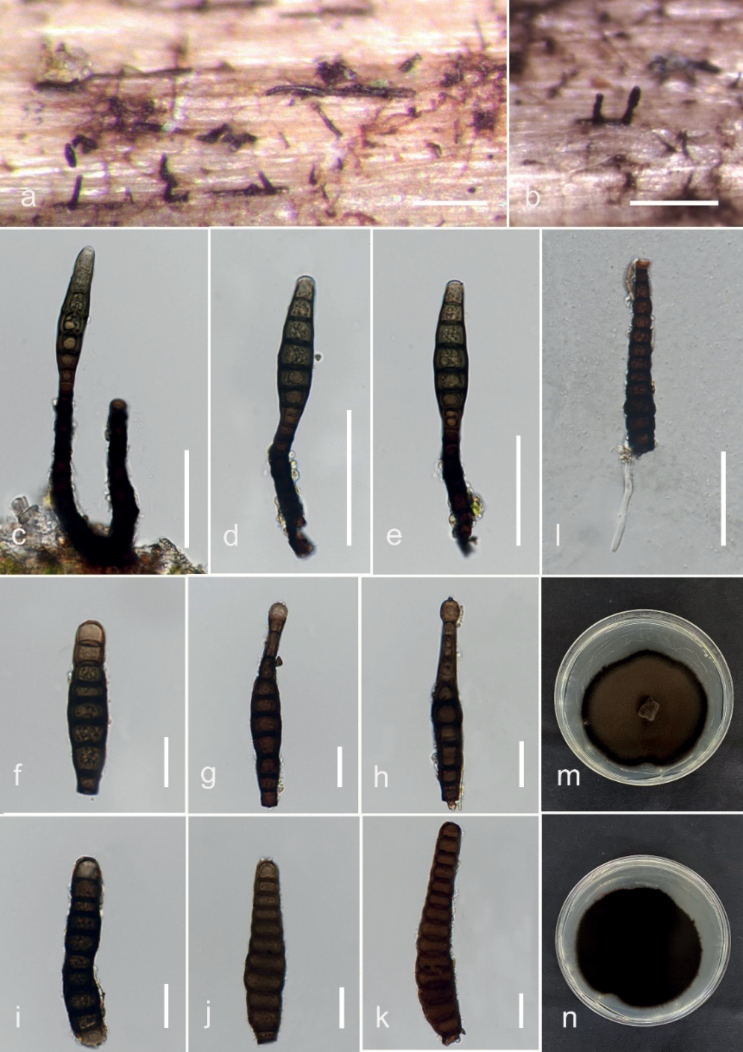
*Kirschsteiniotheliatectonae* (MFLUCC 23–0271, new record) **a, b** colonies on wood **c–e** conidiophores with conidia and conidiogenous cells **f–k** conidia **l** germinated conidium **m, n** culture on PDA (front and reverse). Scale bars: 100 μm (**a, b**); 50 μm (**c–e, l**); 20 μm (**f–k**).

**Figure 7.**
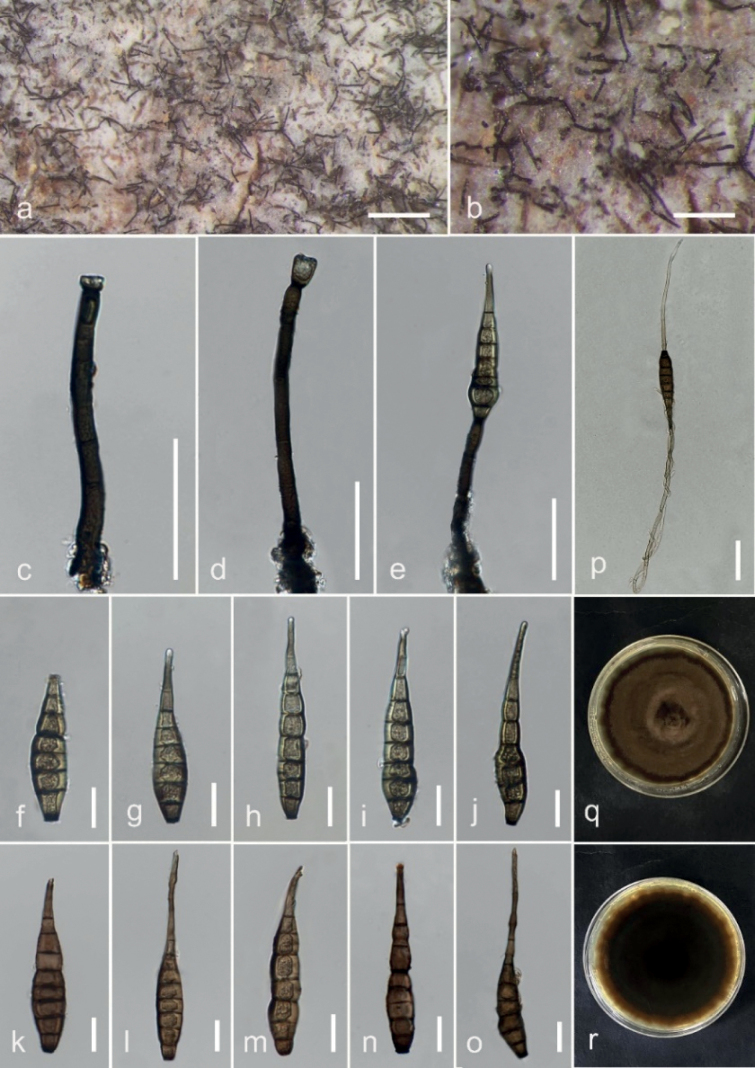
*Kirschsteiniotheliaxishuangbannaensis* (MFLUCC 23–0273, new record) **a, b** colonies on wood **c, d** conidiophores and conidiogenous cells **e** conidiophore with conidium **f–o** conidia **p** germinated conidium **q, r** culture on PDA (front and reverse). Scale bars: 200 μm (**a**); 100 μm (**b**); 50 μm (**c, d, p**); 30 μm (**e**); 20 μm (**f–o**).

Four strains (MFLUCC 23–0277, MFLUCC 23–0270 and MFLUCC 23–0275 and MFLUCC 23–0276) clustered in three independent lineages (Fig. 1). MFLUCC 23–0277 clustered sister to *K.septemseptata* (MFLU 21–0126) with 100% Maximum Likelihood bootstrap support (MLBS) and 1.00 Bayesian posterior probabilities (BYPP) support, while MFLUCC 23–0270 grouped as a sister of *K.emarceis*MFLU 10–0037, but with only 16% MLBS, 0.63 BYPP support, while MFLUCC 23–0275 and MFLUCC 23–0276 clustered with *K.lignicola*MFLUCC 10–0036 with 84% MLBS, 1.00 BYPP support. The other strains clustered with the known species *K.tectonae* (MFLUCC 23–0272 and MFLUCC 23–0271) and *K.xishuangbannaensis* (MFLUCC 23–0273 and MFLUCC 23–0274) with 71% MLBS and 72 MLBS/0.98 BYPP support, respectively. Based on the result of morphological evidence (Figs 2–7), three new species (*K.zizyphifolii*, *K.inthanonensis* and *K.saprophytica*) are proposed, along with the two new host records for *K.xishuangbannaensis* and *K.tectonae*.

### ﻿Taxonomy

#### ﻿ Kirschsteiniothelia inthanonensis

Taxon classificationFungiKirschsteiniothelialesPleosporomycetidae

J. Louangphan & Gomes de Farias sp. nov.

1C554F6B-B53F-5B3A-9FF6-55364AB2D871

Index Fungorum number: IF901384

Facesoffungi Number: FoF14982

Fig. 3

##### Etymology.

The name refers to the location “Doi Inthanon” where the holotype was collected.

##### Holotype.

MFLU 23–0420

##### Description.

Saprobic on decaying wood. ***Sexual morph***: Not observed. ***Asexual morph***: Hyphomycetes. Colonies on the host substrate are superficial, effuse, long hairy, fascicular, scattered, dark brown to black. Mycelium superficial and immersed, composed of branched, septate, pale brown and smooth hyphae. Conidiophores 611–1549 × 2.5–6.6 μm (*x̄* = 1070 × 4.1 μm, n = 20), macronematous, synnematous, compact fasciculate, straight to flexuous, brown to dark brown, branched at the apex, multi-septate, thick and smooth-walled. Conidiogenous cells 15–45 × 6.7–10.4 μm (*x̄* = 24.3 × 8 μm, n = 20), monotretic to polytretic, calyciform, integrated, discrete, terminal, darkened at the apex, proliferating portion, brown, 2–4 septate. Conidia 24–230 × 5.7–14.3 μm (*x̄* = 101 × 9 μm, n = 15), acrogenous, solitary, obclavate, rostrate, straight or curved, truncate at base, grey to brown, pale at apex, partly tapering towards and rounded at the apex, 2–10– euseptate, smooth-walled.

##### Culture characteristics.

Conidia germinated on PDA within 48 hours. Germ tubes germinated from end cell. Colony, reaching 30–35 mm diam. after one month at room temperature, circular form, flat, undulate edges, dense velvety surface, dark green on the surface, white mycelium on the tip, dark in reverse with dark green margin.

##### Material examined.

Thailand, Chiang Mai, Chom Thong, Doi Inthanon National Park, on twigs of *Quercusoleoides*, 30 November 2022, Veenavee Silva, DIFWS5-01 (MFLU 23–0420, holotype), ex-type living culture MFLUCC 23–0277.

##### Notes.

*Kirschsteiniotheliainthanonensis* (MFLUCC 23–0277) resembles *K.septemseptatum* and *K.nabanheensis* in having septate, cylindrical conidiophores with branches near apex, integrated, terminal conidiogenous cells and solitary, obclavate, septate conidia without mucilaginous sheaths. However, *K.inthanonensis*MFLUCC 23–0277 has longer and smaller conidiophores than *K.septemseptatum* and *K.nabanheensis* (611–1549 μm vs. 250–580 μm and 320–588 μm) and (2.5–6.6 μm vs. 6.5–14.5 μm and 8–12 µm), respectively and elongated conidia (Jayawardena et al. 2022; Liu et al. 2023). In addition, our phylogenetic analyses show that *K.inthanonensis* forms an independent branch with 100% MLBS and 1.00 BYPP support. BLASTn base pair comparisons between *K.inthanonensis* (MFLUCC 23–0277) and *K.septemseptatum* (MFLU 21–0126) show 95% similarity of ITS (479/504, 6 gaps), 99% similarity of LSU (844/853, no gaps) and 99% similarity of SSU (787/789, 2 gaps). *Kirschsteiniothelianabanheensis* (HJAUP C2004) shows 94% similarity of ITS (483/513, 7 gaps), 99% similarity of LSU (540/547, no gaps) and 98% similarity of SSU (864/883, no gaps). Based on these data, we introduce *K.inthanonensis* as a new species.

#### ﻿ Kirschsteiniothelia saprophytica

Taxon classificationFungiKirschsteiniothelialesPleosporomycetidae

O. Karimi, V. Silva & Gomes de Farias sp. nov.

85CBCEFC-99F5-56DA-9CF9-16E19025D37A

Index Fungorum number: IF561030

Facesoffungi Number: FoF14983

Figs 4 , 5

##### Etymology.

The species epithet refers to the saprobic life mode of the fungus.

##### Holotype.

MFLU 23–0419

##### Description.

Saprobic on dead wood of undetermined host. ***Sexual morph***: Ascomata 146.7–72.26 µm diam., superficial, solitary, globose to subglobose, dark brown to black. Pseudoparaphyses 1.2–2.7 µm wide (*x̄* = 1.9, n = 20), hyaline, branched, filiform, abounded. Asci 68–125 × 18–23 µm (*x̄* = 101 × 20 µm, n = 10), bitunicate, 8-spored, cylindrical-claviform, sessile or short pedicellate. Ascospores 13–25 (–40) × 7–11 (–14) µm (*x̄*= 24 × 9.8 µm, n = 25), ellipsoid, upper cell broader than lower cell, pale brown to dark brown, 1-septate, guttulate, smooth-walled. ***Asexual morph***: Hyphomycetous. Colonies on host gregarious. Conidiophores 90–216 × 8–12 µm (*x̄* = 165 × 10.6 µm, n = 10), macronematous, mononematous, cylindrical, straight to flexuous, branched, dark brown, multi-septate, constricted at the septa. Conidiogenous cells 6.7–35 × 5–15 µm (*x̄* = 17 × 10 µm, n = 10), holoblastic, monoblastic, terminal, cylindrical, brown to dark brown. Conidia 36–69 × 19–35 µm (*x̄* = 55 × 27 µm, n = 15), cylindrical rounded at ends, 2–3-septa, dark brown to black, smooth-walled.

##### Culture characteristics.

Ascospores germinating on PDA within 24 hours. Colonies growing on PDA 16.8 mm diam. at room temperature after 38 days and on MEA 24 mm after 12 days. Mycelium on PDA superficial to immerse, dark olivaceous to dark brown on the top, reverse dark brown to black. Conidia germinating on PDA within 48 h. Colonies growing on PDA 17 mm diam. at room temperature after 16 days. Mycelium superficial to immerse, dark olivaceous to dark brown on the top, reverse dark brown to black.

##### Material examined.

Thailand, Mae Fah Luang University, Chiang Rai, on dead wood of unidentified host, 20 October 2022, V. Silva, V020 (MFLU 23–0419, holotype), ex-type living culture MFLUCC 23–0275 and MFLUCC 23–0276.

##### Notes.

Our collection (MFLUCC 23–0275) shares similar general characteristics to the type strain *Kirschsteiniothelialignicola* (MFLUCC 10–0105), such as spherical and dark pigmented ascomata, cylindrical to claviform asci, ellipsoidal septate ascospores and cylindrical with brown conidia (Boonmee et al. 2012). However, our collection differs from *K.lignicola* in having shorter asci (68–125 × 18–23 vs. 107–163.3 × 19–28.5 µm), with shorter pedicels (5–6 vs. 14.5–24 µm), shorter conidiophores (90–216 × 8–12 vs. 287–406 × 11–13 µm) and 2–3 transverse septa. Phylogenetically, our isolate clustered with *K.lignicola* with 84% MLBS, 1.00 BYPP. The pairwise base comparisons of the ITS and LSU sequences between *K.saprophytica* and *K.lignicola* showed identities of 93.08% (484/520, 10 gaps) and 91.18% (806/884, 4 gaps), respectively. Based on these differences, we introduce *K.saprophytica* as a new species.

#### ﻿ Kirschsteiniothelia zizyphifolii

Taxon classificationFungiKirschsteiniothelialesPleosporomycetidae

N. Afshari & Gomes de Farias sp. nov.

CE0B06F8-E6CB-5E0E-872D-4B01499839AC

Index Fungorum number: IF901382

Facesoffungi Number: FoF14981

Fig. 2

##### Etymology.

“*zizyphifolii*” refers to the host species on which the fungus was found.

##### Holotype.

MFLU 23–0415

##### Description.

Saprobic on *Nayariophytonzizyphifolium* (Malvaceae) woody litter in terrestrial habitat. ***Sexual morph***: Not observed. ***Asexual morph***: Hyphomycetes. Colonies on the substratum are superficial, effuse, dark brown to black and hairy. Mycelia superficial, composed of septate, branched, smooth-walled, dark brown hyphae. Conidiophores 287–444.5 × 10.3 –17 (–19.7) μm (*x̄* = 358.5 × 13.4 μm, n = 15), macronematous, mononematous, erect, with several short branches near the apex, irregular, solitary, cylindrical, flexuous, sometimes slightly straight, dark brown to black, paler towards the apex, septate, smooth-walled. Conidiogenous cells 11–20.4 × 5.8–10.6 μm (*x̄* = 14.6 × 7.6 μm, n = 25), tretic, occasionally percurrent, integrated, terminal or intercalary, cylindrical or doliiform, brown, smooth-walled. Conidia (29.5–) 37.6–46.5 × 13.5–19 μm (*x̄* = 43 × 16 μm, n = 20), acrogenous, solitary, cylindrical to rarely clavate, rounded at the apex, straight or moderately curved, brown dark to brown, 2–3-septate, constricted and pigmented at the septa, smooth-walled.

##### Culture characteristics.

Ascospores germinating on PDA within 24 hours, reaching up to 30 mm diam. after one week at room temperature. Germ tubes germinated from both end cells. Colony dense, circular, velvety, narrow towards the edge, from front, grey at centre, black towards edge, from reverse, black.

##### Material examined.

Thailand, Chiang Rai, Mae Fa Luang, Doi Tung Forest, on dead wood of *Nayariophytonzizyphifolium*, 26 March 2022, N. Afshari 1C1T2R4b (MFLU23–0415, holotype), ex-type living culture MFLUCC 23–0270.

##### Notes.

*Kirschsteiniotheliazizyphifolii* (MFLUCC 23–0270) resembles *K.lignicola* (MFLUCC 10–0036) and *K.emarceis* (MFLU 10–0037) in having erect and branched conidiophores with apical dark brown conidia. However, it differs from *K.lignicola* in the sizes of conidiophores and conidia. Furthermore, BLASTn search of ITS and LSU sequences showed that *K.zizyphifolii* was closest to *K.emarceis* with similarity values of 90% (472/522, 12 gaps) and 84% (708/842, 22 gaps), respectively. Furthermore, our isolate (MFLUCC 23–0270) was close to *K.lignicola* (MFLUCC 10–0036) with similarity values of 89% (ITS = 474/532, 19 gaps), 99% (LSU = 844/853, 2 gaps) and 99% (SSU = 643/648, 2 gaps). Based on these phylogenetic data, we introduce *K.zizyphifolii* as a new species.

#### ﻿ Kirschsteiniothelia tectonae

Taxon classificationFungiKirschsteiniothelialesPleosporomycetidae

Doilom, Bhat & K.D. Hyde, 2016

68E7C110-D340-564C-8CDB-39E6A5F18A08

Index Fungorum number: IF551992

Facesoffungi Number: FoF01883

Fig. 6

##### Description.

Saprobic on *Microcospaniculata* (Malvaceae) woody litter in terrestrial habitats. ***Sexual morph***: Not observed. ***Asexual morph***: Hyphomycetes. Colonies on the substrate, hairy, superficial, dark brown, scattered, partially grouped. Conidiophores 59–90 × 8.6–12 μm (*x̄* = 75 × 10.7 μm, n = 10), superficial, simple, macronematous, mononematous, cylindrical, straight to slightly curved, branched or unbranched, septate, dark brown to black. Conidiogenous cells 7–9.4 × 6–7.3 μm (*x̄* = 8 × 6.7 μm, n = 5), monoblastic, determinate, integrated, terminal. Conidia 62.5– 133 × 11 – 18.5(–21) μm (*x̄* = 94 × 16 μm, n = 30), cylindrical-obclavate, elongate, straight to slightly curved, rounded being slightly paler at the apex, obconically truncate at the base, 7–12–septa, olivaceous green to brown, smooth–walled.

##### Culture characteristics.

Conidia germinating on PDA within 24 hours, reaching up to 15–20 mm diam. after one week at room temperature. Germ tubes generated from basal cells. Colony on PDA, dense, circular, flat or effuse, velvety, from front brown at the centre and black at the edge, from reverse, dark brown.

##### Material examined.

Thailand, Chiang Rai, Mae Fa Luang, Doi Tung, on dead wood of *Microcospaniculata*, 6 June 2022, N. Afshari 3C2T3R5 (MFLU 23–0416), living culture MFLUCC 23–0272. On dead wood of *Dalbergiacana*, 3 March 2022, N. Afshari 4C1T2R3 (MFLU 23–0417), living culture MFLUCC 23–0272.

##### Known distribution.

Thailand (Li et al. 2016; this study)

##### Known hosts.

*Tectonagrandis* (Li et al. 2016), *Microcospaniculata* and *Dipterocarpusalatus* (this study)

#### ﻿ Kirschsteiniothelia xishuangbannaensis

Taxon classificationFungiKirschsteiniothelialesPleosporomycetidae

R.F. Xu & Tibpromma

14EA46AC-3DB8-532C-B62E-4C83AFE5936E

Index Fungorum number: IF559433

Facesoffungi Number: FoF12758

Fig. 7

##### Description.

Saprobic on *Microcospaniculata* (Malvaceae) woody litter in terrestrial habitats. ***Sexual morph***: Not observed. ***Asexual morph***: Hyphomycetes. Colonies effuse on the substrate, hairy, solitary or scattered, dark brown. Conidiophores 135–178 × 7.7–11 μm (*x̄* = 151 × 9 μm, n = 10), macronematous, straight to curved, solitary, brown, slightly larger at base, narrowing towards apex, septate. Conidiogenous cells 14.4–27.4 × 7.8–11 μm (*x̄* = 22 × 10 μm, n = 10), holoblastic, monoblastic, integrated, smooth, terminal, determinate, cylindrical or lageniform, brown. Conidia 70–141 × 14.5–19 μm (*x̄* = 100 × 17 μm, n = 20), solitary, acrogenous, obclavate, rostrate, straight or slightly curved, truncate at the base, olivaceous green to brown, subhyaline at the apex, 5–10-septate, large guttulate.

##### Culture characteristics.

Conidia germinating on PDA within 24 hours reaching up to 2 cm diam. after one week at room temperature. Germ tubes generated from both end cells. Colony on PDA, dense, circular, flat or effuse, velvety, from front, brown at the centre and dark brown at edge, from reverse, black to pale brown radiating.

##### Material examined.

Thailand, Chiang Rai, Mae Fa Luang, Doi Tung, on dead wood of *Microcospaniculata*, 6 June 2022, N. Afshari 3C2T1R1, living culture MFLUCC 23–0273. On dead wood of *Dipterocarpusalatus*, 27 September 2022, N. Afshari 2C3T1R3c (MFLU 23–0418), living culture MFLUCC 23–0274.

##### Known distribution.

China (Xu et al. 2023), Thailand (this study).

##### Known hosts.

*Heveabrasiliensis* (Xu et al. 2023), *Microcospaniculata* and *Dalbergiacana* (this study).

## ﻿Discussion

This study introduces three new species and new host records of *Kirschsteiniothelia* from dead wood from Chiang Rai Province, Thailand, based on morphological and molecular analyses (Figs 1–7). *Kirschsteiniothelia* species have been found almost worldwide, including in the United States of America (Hawksworth 1985; Hyde 1997; Wang et al. 2004; Su et al. 2016), Iran (Mehrabi et al. 2017), Switzerland (Hawksworth 1985; Wang et al. 2004), Thailand (Boonmee et al. 2012; Li et al. 2016; Bao et al. 2018; Hyde et al. 2018; Sun et al. 2021; Jayawardena et al. 2022), South Africa (Marincowitz et al. 2008), China (Chen et al. 2006; Su et al. 2016; Bao et al. 2018; Liu et al. 2023; Yang et al. 2023; Xu et al. 2023), Canada (Hawksworth 1985), Italy (Wang et al. 2004), Spain (Rodríguez-Andrade et al. 2019) and India (Bao et al. 2018). Most of the species (*K.acutispora*, *K.chiangmaiensis*, *K.crustacea*, *K.emarceis*, *K.extensa*, *K.lignicola*, *K.phoenicis*, *K.rostrata*, *K.septemseptata*, *K.spatiosa*, *K.tectonae* and *K.thailandica*) have been reported from Thailand (Boonmee et al. 2012; Li et al. 2016; Bao et al. 2018; Hyde et al. 2018; Sun et al. 2021; Jayawardena et al. 2022), representing more than 25% of the species in this genus. Our results expand the knowledge of the diversity of this genus, especially in Thailand.

This genus is also prone to be highly speciose, given the recent introduction of ten new species (Jayawardena et al. 2022; Hyde et al. 2023; Liu et al. 2023; Louangphan et al. 2023 (under review); Xu et al. 2023). With the introductions of the present study (*K.inthanonensis*, *K.saprophytica*, *K.paniculata* and *K.zizyphifolii*), 32.5% of the species will have been introduced within two years, mainly as saprobes in woody litter. Besides, most *Kirschsteiniothelia* species have been reported from terrestrial environments, with only a few (*K.cangshanensis*, *K.fluminicola* and *K.rostrata*) reported from freshwater habitats (Bao et al. 2018). Their ecological significance also relies on their ability to infect humans (Nishi et al. 2018; Guegan et al. 2021). This demonstrates the potential for further discoveries on the diversity and lifestyles within *Kirschsteiniothelia*. Thus, exploring its diversity, especially in woody litter in protected environments and other tropical areas, will reveal the vast diversity within Kirschsteiniotheliaceae. For example, frequent incursions into fungal diversity have established Thailand as a hotspot for its diversity (Hyde et al. 2018).

Furthermore, *Kirschsteiniothelia* species appear to not have host specificity, as from our results, the same species were found associated with different hosts: *K.xishuangbannaensis*, previously reported from dead branches of *Heveabrasiliensis* (Xu et al. 2023), was recorded from *Microcospaniculata* (MFLUCC 23–0273) and *Dipterocarpusalatus* (MFLUCC 23–0274); *K.paniculata* was isolated from *Microcospaniculata* (MFLUCC 23–0271) and *Dalbergiacana* (MFLUCC 23–0272). In this regard, the host-specificity or host-recurrence of saprobic fungi has been discussed over the last two decades (Hooper et al. 2000; Zhou and Hyde 2001; Santana et al. 2005; Kodsueb et al. 2008; Tennakoon et al. 2022). However, saprotrophs seem to be less host-specific when compared with other trophic modes (Zhou and Hyde 2001). This may be because different hosts have different chemical compositions, which may affect the fungi of a particular species (Hyde et al. 2007). This hypothesis suggests that woody litter may harbour many species yet to be discovered (Kodsueb et al. 2008).

A combined approach should be employed to resolve the taxonomic placement of new species in this genus. This approach should include at least molecular phylogeny and morphological characters (Chethana et al. 2021; Maharachchikumbura et al. 2021). It should also include the linking of sexual and asexual morphologies, which are important factors in the taxonomy of Ascomycota, as pleomorphism can bias the morphological characters (Maharachchikumbura et al. 2021). However, only a few of the 39 *Kirschsteiniothelia* species, specifically *K.atra* and *K.recessa* (Hawksworth 1985) and *K.lignicola* and *K.emarceis* (Boonmee et al. 2012), are known from both their sexual and asexual morphs.

The findings of this study underscore the importance of integrating multiple types of evidence for the identification and classification of fungal species and they demonstrate the potential for further discoveries within *Kirschsteiniothelia*. The discovery of new species and host records has significant implications for our understanding of the ecological roles and interactions of this genus. In particular, identifying new host records provides valuable insights into the host range and specificity of *Kirschsteiniothelia* species, which may help elucidate the mechanisms underlying these interactions. Further research is necessary to fully explore the ecological significance of these findings and determine the potential impacts of *Kirschsteiniothelia* species on their hosts and ecosystems.

## Supplementary Material

XML Treatment for Kirschsteiniothelia inthanonensis

XML Treatment for Kirschsteiniothelia saprophytica

XML Treatment for Kirschsteiniothelia zizyphifolii

XML Treatment for Kirschsteiniothelia tectonae

XML Treatment for Kirschsteiniothelia xishuangbannaensis

## ﻿Additional information

### Author ORCIDs

Antonio Roberto Gomes de Farias https://orcid.org/0000-0003-4768-1547

Veenavee S. Hittanadurage Silva https://orcid.org/0000-0001-8921-1370

Omid Karimi https://orcid.org/0000-0001-9652-2222

Saranyaphat Boonmee https://orcid.org/0000-0001-5202-2955

### Data availability

All of the data that support the findings of this study are available in the main text.
